# Improvements in Neoplasm Classification in the International Classification of Diseases, Eleventh Revision: Systematic Comparative Study With the Chinese Clinical Modification of the International Classification of Diseases, Tenth Revision

**DOI:** 10.2196/52296

**Published:** 2024-03-08

**Authors:** Yicong Xu, Jingya Zhou, Hongxia Li, Dong Cai, Huanbing Zhu, Shengdong Pan

**Affiliations:** 1 Medical Records Room, Department of Medical Administration The Second Affiliated Hospital, Zhejiang University School of Medicine Hangzhou China; 2 Department of Medical Records Peking Union Medical College Hospital, Chinese Academy of Medical Sciences and Peking Union Medical College Beijing China; 3 Collaborating Center for the WHO Family of International Classifications in China Beijing China; 4 Department of Gastroenterology The Second Affiliated Hospital, Zhejiang University School of Medicine Hangzhou China; 5 Department of Hepatobiliary and Pancreatic Surgery The Second Affiliated Hospital, Zhejiang University School of Medicine Hangzhou China; 6 Department of Medical Administration The Second Affiliated Hospital, Zhejiang University School of Medicine Hangzhou China

**Keywords:** Chinese Clinical Modification of the International Classification of Diseases, Tenth Revision, ICD-10, ICD-10-CCM, ICD-11, improvement, International Classification of Diseases, Eleventh Revision, International Classification of Diseases, Tenth Revision, International Classification of Diseases, neoplasm, transition

## Abstract

**Background:**

The International Classification of Diseases, Eleventh Revision (ICD-11) improved neoplasm classification.

**Objective:**

We aimed to study the alterations in the ICD-11 compared to the Chinese Clinical Modification of the International Classification of Diseases, Tenth Revision (ICD-10-CCM) for neoplasm classification and to provide evidence supporting the transition to the ICD-11.

**Methods:**

We downloaded public data files from the World Health Organization and the National Health Commission of the People’s Republic of China. The ICD-10-CCM neoplasm codes were manually recoded with the ICD-11 coding tool, and an ICD-10-CCM/ICD-11 mapping table was generated. The existing files and the ICD-10-CCM/ICD-11 mapping table were used to compare the coding, classification, and expression features of neoplasms between the ICD-10-CCM and ICD-11.

**Results:**

The ICD-11 coding structure for neoplasms has dramatically changed. It provides advantages in coding granularity, coding capacity, and expression flexibility. In total, 27.4% (207/755) of ICD-10 codes and 38% (1359/3576) of ICD-10-CCM codes underwent grouping changes, which was a significantly different change (*χ*^2^_1_=30.3; *P*<.001). Notably, 67.8% (2424/3576) of ICD-10-CCM codes could be fully represented by ICD-11 codes. Another 7% (252/3576) could be fully described by uniform resource identifiers. The ICD-11 had a significant difference in expression ability among the 4 ICD-10-CCM groups (*χ*^2^_3_=93.7; *P*<.001), as well as a considerable difference between the changed and unchanged groups (*χ*^2^_1_=74.7; *P*<.001). Expression ability negatively correlated with grouping changes (*r*=–.144; *P*<.001). In the ICD-10-CCM/ICD-11 mapping table, 60.5% (2164/3576) of codes were postcoordinated. The top 3 postcoordinated results were specific anatomy (1907/3576, 53.3%), histopathology (201/3576, 5.6%), and alternative severity 2 (70/3576, 2%). The expression ability of postcoordination was not fully reflected.

**Conclusions:**

The ICD-11 includes many improvements in neoplasm classification, especially the new coding system, improved expression ability, and good semantic interoperability. The transition to the ICD-11 will inevitably bring challenges for clinicians, coders, policy makers and IT technicians, and many preparations will be necessary.

## Introduction

The World Health Organization (WHO) adopted the International Classification of Diseases, Tenth Revision (ICD-10) in May 1990 [1]. The ICD-10 has been widely used in over 120 countries over the past 30 years. In multiple countries, expansions of the ICD-10, such as the Chinese Clinical Modification of the International Classification of Diseases, Tenth Revision (ICD-10-CCM), based on the second edition [2], have been developed to meet specific requirements. The ICD-10-CCM has been used for national performance assessment and medical insurance payment in public hospitals. Given the advances in medical knowledge and health information, the WHO started working on the International Classification of Diseases, Eleventh Revision (ICD-11) in 2007 [3], and it was expected to be implemented by WHO member countries starting in January 2022.

The most critical changes in the ICD-11 were the establishment of a semantic knowledge base and the reconstruction of the coding system. It introduced the Foundation Component, the Common Ontology, and linearization, as well as the new concepts of precoordination and postcoordination [3-5]. The chapter on neoplasms has also changed. It adds crucial morphology to precoordination, having the highest proportion (98.9%) and the most dimensions (3.5) of postcoordination. Cancer has been one of the top 3 causes of death in China since 2005 [6]; thus, the coding system of the ICD-11 will significantly impact cause of death reporting, cancer registration, and disease diagnosis records. Stakeholders need to have a good understanding of the classification of neoplasms in the ICD-11.

This study analyzes the changes between the ICD-10-CCM and ICD-11 in terms of coding features, classification features, and expression features in neoplasm classification and hopes to provide evidence supporting the transition in China.

## Methods

### Ethical Considerations

According to the Measures for Ethical Review of Human Life Science and Medical Research issued by the National Health Commission of the People's Republic of China, this study utilized public data and did not involve human subjects, and thus, the requirement of ethical permission was waived. All examples were constructed and neither correspond to real clinical cases nor to any datasets.

### Materials

The following 3 existing, publicly available files were used in this paper: (1) ICD-10/ICD-11 mapping tables [7], (2) ICD-11 simple tabulation [8], and (3) the second revision of the ICD-10-CCM [9].

The first 2 files were downloaded from the WHO website, and the ICD-10-CCM was released by the National Health Commission of the People’s Republic of China.

### Research Methods

The ICD-10-CCM neoplasm codes were manually recoded by the ICD-11 coding tool [10] based on the International Classification of Diseases, Eleventh Revision, Mortality and Morbidity Statistics (ICD-11 MMS) codes to generate an ICD-10-CCM/ICD-11 mapping table for neoplasms. The 3 existing files and the ICD-10-CCM/ICD-11 mapping table were used to analyze ICD-11 features, namely, the coding structure, coding capacity, grouping changes, expression ability, expression flexibility, and the expression of postcoordination in the neoplasm classification.

### Mapping ICD-10-CCM to ICD-11 Codes

Due to the homology of the morphology section between the ICD-10 and ICD-11, mapping was performed for only the topography codes. To ensure the accuracy of the results, manual recoding was independently implemented by 2 authors who both had more than 10 years of coding experience and had received ICD-11 training. Inconsistent results were resolved by consulting a senior coder from the Collaborating Center for the WHO Family of International Classifications in China.

### Statistical Standards

#### Leaf Codes

The ICD-10, ICD-10-CCM, and ICD-11 MMS codes that can be used at the lowest level are called leaf codes. All statistical analyses were based on the leaf codes.

#### Coding Capacity

This capacity involves the number of leaf codes that can be used in actual coding.



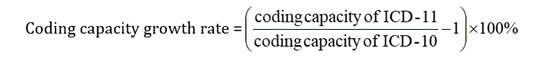



#### Grouping Changes

Grouping was based on the block structure of the ICD. The equivalent groups included ICD-10 group 1 (malignant neoplasms) and ICD-11 group 3 (malignant neoplasms, except for lymphoid, hematopoietic, central nervous system, or related tissues), ICD-10 group 2 (in situ neoplasms) and ICD-11 group 4 (in situ neoplasms, except for lymphoid, hematopoietic, central nervous system, or related tissues), and ICD-10 group 3 (benign neoplasms) and ICD-11 group 5 (benign neoplasms, except for lymphoid, hematopoietic, central nervous system, or related tissues). If an ICD-10/ICD-10-CCM leaf code was not classified into the equivalent ICD-11 group, it was considered to have undergone a grouping change.

#### Expression Ability

For each ICD-10-CCM code, we identified the best-matching ICD-11 MMS leaf code. When all the clinical details in the diagnosis were expressed without redundant information, the code was considered fully represented. This study also defined the synonyms contained in the Foundation Component as a full representation.

### Statistical Analysis

All data were analyzed with SPSS (version 25.0, IBM). The changes in coding capacity, groups, and expression ability between the ICD-10 and ICD-11 were described as rates and percentages. The chi-square test was used to determine the difference in grouping changes when mapped to the ICD-11 between the ICD-10 and ICD-10-CCM. The chi-square test was also used to analyze the difference in full expression ability among the 4 ICD-10-CCM groups, and the Bonferroni method was used for pairwise comparisons. The chi-square test was used to analyze the difference in full expression ability between the changed and unchanged groups mapped to the ICD-11. The φ correlation coefficient was used to analyze the correlation between expression ability and grouping changes. Statistical significance was set at *P*<.05.

## Results

### Coding Features

#### Coding Structure

Chapter 2 in both the ICD-10 and ICD-11 addresses neoplasms, and the codes in both versions are alphanumeric but have different structures. The ICD-10 codes for neoplasms consist of topography codes and morphology codes. The topography codes range from C00 to D48. Except for leaf codes, the coding range, categories, and subcategories of the ICD-10-CCM are the same as those of the ICD-10. The ICD-10 morphology codes consist of 5 digits. The first 4 digits identify the histological type of the neoplasm, and the fifth digit, following a slash (/), indicates its behavior. In the ICD-10-CCM, the morphology codes consist of 6 digits, with a fifth number before the slash.

In contrast, the coding structure of the ICD-11 MMS codes has dramatically changed. It is composed of stem codes and extension codes that are connected by an ampersand (&). The precoordinated stem codes consist of sites and essential morphology types. The newly added chapter on extension codes addresses other morphology types and greater site specificity [11], as well as stage, grading, laterality, and the diagnostic method. These codes can be used for postcoordination. The stem codes range from 2A00 to 2F9Z. The extension codes for morphology are 6-digit codes composed of letters and numbers, starting with the letter *X*. Examples of the ICD-10 and ICD-11 complete neoplasm codes are shown in Table 1.

**Table 1:** Examples of the International Classification of Diseases, Tenth Revision (ICD-10), Chinese Clinical Modification of the International Classification of Diseases, Tenth Revision (ICD-10-CCM), and International Classification of Diseases, Eleventh Revision (ICD-11) complete neoplasm codes.

Neoplasm	ICD-10		ICD-10-CCM		ICD-11	
	Topography code	Morphology code	Topography code	Morphology code	Stem code	Extension codes
Adenocarcinoma of the common bile duct, stage III, diagnosis confirmed by histology	C24.0	8140/3	C24.003	81400/3	2C15.0	&XS6H &XY9Q

In the ICD-10, C24.0 represents a malignant neoplasm of the extrahepatic bile duct, and 8140/3 represents adenocarcinoma. In the ICD-10-CCM, C24.003 represents a malignant neoplasm of the common bile duct, and 81400/3 represents adenocarcinoma. In the ICD-11, 2C15.0 represents adenocarcinoma of the distal bile duct, XS6H represents stage III, and XY9Q represents a diagnosis confirmed by histology. We used the the ICD-11 coding tool website [10].

#### Coding Capacity

Categories, subcategories, and leaf codes can be used for statistics, but only leaf codes can be used for actual coding. In the ICD-10, chapter 2 includes 759 leaf codes [7]. In the ICD-10-CCM, the topography codes are basically expanded by refining the sites, with 3634 leaf codes for neoplasms. Taking malignant neoplasm of the nasal cavity (C30.0) as an example, the ICD-10-CCM contains 5 additional leaf codes, such as a malignant neoplasm of nasal cartilages (C30.001) and a malignant neoplasm of the nasal concha (C30.002).

In the ICD-11, chapter 2 includes 1037 leaf codes [8]. Compared with the ICD-10, the number of ICD-11 leaf codes was expanded by 36.6%, which is conducive to better granularity of statistics and classification.

### Classification Feature

The ICD-11 has readjusted the neoplasm groups. In the ICD-10, chapter 2 was divided into 4 groups: malignant neoplasms, in situ neoplasms, benign neoplasms, and neoplasms of uncertain or unknown behavior. In the ICD-11, chapter 2 was increased to 7 groups (Table 2). The newly added ICD-11 group 1 includes all neoplasms of the brain and central nervous system, regardless of behavior, and the newly added ICD-11 group 2 includes all hematopoietic and lymphoid tissues. The ICD-10 group 4 was split into 2 separate groups: ICD-11 group 6 (neoplasms of uncertain behavior) and ICD-11 group 7 (neoplasms of unknown behavior).

**Table 2:** Grouping changes between the International Classification of Diseases, Tenth Revision (ICD-10) and the International Classification of Diseases, Eleventh Revision (ICD-11).

Classification system		ICD-11								
		Group 1	Group 2	Group 3	Group 4	Group 5	Group 6	Group 7	Other chapters	No mapping
**ICD-10 codes, n**										
	Group 1	23^a^	91^a^	352	N/A^b^	N/A	N/A	N/A	8^a^	2
	Group 2	N/A	N/A	N/A	57	N/A	N/A	N/A	N/A	N/A
	Group 3	11^a^	N/A	1^a^	N/A	146	N/A	N/A	1^a^	N/A
	Group 4	11^a^	18^a^	N/A	N/A	N/A	48^a^	51^a^	2^a^	2
**ICD-10-CCM codes, n**										
	Group 1	93^a^	499^a^	1252	N/A	N/A	N/A	N/A	17^a^	3
	Group 2	N/A	N/A	N/A	169	N/A	N/A	N/A	N/A	N/A
	Group 3	118^a^	N/A	1^a^	N/A	796	N/A	N/A	3^a^	N/A
	Group 4	85^a^	60^a^	N/A	N/A	N/A	316^a^	149^a^	18^a^	55

^a^Indicates grouping changes. The ICD-10 and ICD-10-CCM no mapping codes were not included in the statistical analysis of this study. Hence, 755 ICD-10 codes and 3576 ICD-10-CCM codes were used for percentage and chi-square analyses.

^b^N/A: not applicable.

In total, 27.4% (207/755) of ICD-10 codes underwent grouping changes. Among them, 150 codes were migrated by 1 group, 56 by 2 groups, and one by 3 groups. In the ICD-10-CCM, 38% (1359/3576) leaf codes underwent grouping changes (Table 2). A chi-square test revealed significant differences in grouping changes between the ICD-10 and ICD-10-CCM (*χ*^2^_1_=30.3; *P*<.001).

### Expression Features

#### Expression Ability

Because of the classification changes, 58 of the 3634 ICD-10-CCM codes for neoplasms could not be recoded. In total, 3576 codes were included in the manual recoding study. The results of 2 separate recodings showed that 6% (213/3576) codes were mapped inconsistently. A total of 32 stem codes were inconsistent, and 181 extension codes differed. The 213 codes were all identified after consultation with the senior coder from the Collaborating Center for the WHO Family of International Classifications in China.

The final results showed that 16.6% (594/3576) codes were fully represented without postcoordination, 51.2% (1830/3576) codes were fully characterized with postcoordination, and the remaining 32.2% (1152/3576) codes were only partially described (Table 3). In addition, 7% (252/3576) codes were fully represented when using uniform resource identifiers (URIs).

**Table 3:** Comparison of the International Classification of Diseases, Eleventh Revision (ICD-11) expression ability among the 4 Chinese Clinical Modification of the International Classification of Diseases, Tenth Revision (ICD-10-CCM) groups.

ICD-10-CCM group	Total codes, N	Full representation				Partial representation	
		With PC^a^ (a), n	Without PC (b), n	Codes (n1=a+b), n	Codes (n1/N), %	Codes (n2), n	Codes (n2/N), %
Group 1	1861	935	406	1341	72.1	520	27.9
Group 2	169	106	44	150	88.8	19	11.2
Group 3	918	501	73	574	62.5	344	37.5
Group 4	628	288	71	359	57.2	269	42.8
Total	3576	1830	594	2424	67.8	1152	32.2

^a^PC: postcoordination.

Based on the 4 ICD-10-CCM groups, ICD-11 significantly differed in expression ability (*χ*^2^_3_=93.7; *P*<.001). The Bonferroni method showed that the ICD-11 had the most robust expression ability in the ICD-10-CCM group 2, followed by the ICD-10-CCM group 1, and there was no significant difference between the remaining 2 groups.

Based on whether grouping changes occurred during mapping, 73.1% (1620/2217) ICD-10-CCM codes in the unchanged group and 59.2% (804/1359) ICD-10-CCM codes in the changed group were fully expressed, and the difference was significant (*χ*^2^_1_=74.7; *P*<.001). Expression ability had a negative correlation with grouping changes (*r*=–.144; *P*<.001).

#### Expression Flexibility

The expression flexibility of the ICD-11 is reflected in many aspects. For instance, in the ICD-10, the subcategory “.8” generally describes overlapping neoplasm sites, while in the ICD-11, several methods are used. Specifically, the ICD-11 uses multiple extension codes. Sometimes, only 1 extension code is used, such as XA4YW8 (overlapping sites of the esophagus). Occasionally, the ICD-11 describes this condition through stem codes, such as 2B71.0 (adenocarcinoma of the esophagogastric junction). ICD-11 classifications can also be represented through URIs, for instance, http://id.who.int/icd/entity/419755630 (Kaposi sarcoma of multiple organs).

Expression flexibility is also reflected in the additional option of postcoordination, which can meet the different requirements of most hospitals for clinical phenotype mining. For example, a patient with hepatocellular carcinoma in the left lobe of the liver that causes chronic intermittent cancer pain and tumor anemia would be coded as 2C12.02&XA5766/MG30.10&XT5G/3A71.0. Postcoordination fully expresses the clinical phenotypes and demonstrates the relationship between diseases and clinical phenotypes.

#### Expression of Postcoordination

According to the ICD-10-CCM/ICD-11 mapping table, 60.5% (2164/3576) of codes had postcoordination, and the average amount of postcoordination per code was approximately 0.7. Regarding the type of postcoordination, the proportion of specific anatomy (1907/3576, 53.3%) was the highest, followed by histopathology (201/3576, 5.6%), and the remaining dimensions were basically less than 2% (Table 4).

**Table 4:** Expression of postcoordination in the Chinese Clinical Modification of the International Classification of Diseases, Tenth Revision/International Classification of Diseases, Eleventh Revision (ICD-10-CCM/ICD-11) mapping table.

Number and dimensions of postcoordination		Codes, n (%)
**0**		1412 (39.5)
	No	
**1**		
	Specific anatomy	1750 (48.9)
	Histopathology	176 (4.9)
	Alternative severity 2	60 (1.7)
	Laterality	5 (0.1)
	Course of the condition	3 (0.1)
	Stem codes	3 (0.1)
**2**		
	Specific anatomy/specific anatomy	118 (3.3)
	Specific anatomy/histopathology	17 (0.5)
	Specific anatomy/laterality	8 (0.2)
	Histopathology/alternative severity 2	8 (0.2)
	Alternative severity 2/stem codes	2 (0.1)
**3**		
	Specific anatomy/specific anatomy/specific anatomy	11 (0.3)
	Specific anatomy/specific anatomy/laterality	3 (0.1)

## Discussion

### Principal Findings

The different coding structure of the ICD-11 provides advantages in terms of coding granularity, coding capacity, and expression flexibility. According to the mapping tables, the grouping changes between the ICD-10 and ICD-10-CCM differed (*χ*^2^_1_=30.3; *P*<.001). Meanwhile, neither the ICD-10 group 2 nor the ICD-10-CCM group 2 exhibited group migration when mapped to the ICD-11. In this study, ICD-11 expression ability (67.8%) was slightly higher than that in other studies (60%) [12,13]. Among the 4 ICD-10-CCM groups, there were significant differences in expression ability (*χ*^2^_3_=93.7; *P*<.001), with ICD-10-CCM group 2 having the highest expression ability. The expression ability negatively correlated with grouping changes (*r*=–0.144; *P*<.001).

The expression ability of the ICD-11 was still underestimated. There were many reasons why 1152 ICD-10-CCM codes did not have full representation, some of which can be avoided in actual cases. First, of the 296 ICD-10-CCM codes mapped to the ICD-11 group 1, only 26 (8.8%) codes were fully expressed, which is far below the overall level. Among them, 217 codes cannot be fully expressed because most of the stem codes in ICD-11 group 1 do not include behavior. However, in coding actual cases with morphological types, some stem codes, including behavior, would be used instead of the residual category. Therefore, in actual coding, the expression ability of this group would be higher than that determined in this study.

Second, 714 codes could not be fully expressed due to site classification. Fortunately, 109 codes detailing specific sites may be resolved by refining the value set of extension codes. Examples include the frenulum of the upper lip, the ileocecal valve, and the rectouterine recess. However, there were also some codes for which the classification was different, such as peripheral nervous system neoplasms. This condition could be solved by coding the actual cases. In addition, some words, such as canceration of the gastric stump and cervical stump, need to be addressed.

Third, URIs can supplement the function of the ICD-11 MMS codes, especially for some diseases that cannot be identified by the ICD-11 codes. For instance, URIs (http://id.who.int/icd/entity/1595913346) make classic Kaposi sarcoma classifiable. The new coding system can also make other diseases identifiable [14-16], such as chronic pain and rare diseases. In summary, the ICD-11 has advantages in terms of actual coding and can address the expression needs of neoplasms.

### The ICD-11 Vs the ICD-10

Compared to the ICD-10, the ICD-11 has undergone significant changes from design to use. First, the design purpose of the ICD-11 has changed. For more than a century, the ICD has been the basis for comparable statistics on causes of mortality and morbidity between places and over time. As a statistically friendly classification system, when the ICD-10 is used for clinical term records and diagnosis-related grouping, it cannot meet practical needs, resulting in various expanded versions of the WHO ICD-10 in multiple countries, which often leads to inconsistent statistical standards for data. In contrast, the ICD-11 is a clinically friendly classification system that meets diverse goals beyond mere health statistics, including clinical term records, patient safety and quality, reimbursement, decision support, and more. The ICD-11 is entirely digital, terminology is coded with the coding tool and application programming interface, and it has a semantic knowledge base; these features are beneficial for standardized data collection. In short, the ICD-11 has benefits in terms of obtaining statistics as well as multiaxial coding, coding granularity [4,17,18] and standardization, achieving the integration of terminology and classification. As shown in Table 1, although the ICD-10-CCM codes are expanded and refined at the leaf code level, the capture of clinical details is still weaker compared to the ICD-11 codes.

Second, the design concept of the ICD-11 is different. The ICD-10 is an independent classification system, and the standard terminology set for the ICD-10 has been developed independently. Differently, the ICD-11 has good semantic interoperability with other classification systems through harmonized methods. The Systematized Nomenclature of Medicine-Clinical Terms (SNOMED CT) is considered one of the most comprehensive clinical terminologies in the world [19]. The ICD-11 Foundation Component, which includes semantic network concepts and their relationships, is organized around the Common Ontology from a subset of the SNOMED CT [20,21]. The Common Ontology has been harmonized with ICD text definitions, primarily from the SNOMED CT clinical findings hierarchy (findings, disorders, and disease) and secondarily from other hierarchies (situations, events, social context, and so on) [22]. The rich Foundation Component has approximately 80,000 entries and 40,000 synonyms [5]. The ICD-11 linearizations, including the ICD-11 MMS and the International Classification of Diseases for Oncology (ICD-O), are subsets derived from the Foundation Component. The ICD-11 integrates the morphology section of the ICD-O, ICD-O linearization, and tumor node metastasis classification, and the histopathology codes of the ICD-11 are also compatible with the ICD-O [4]. Additionally, the ICD-11 integrates numerous clinical terminologies from some expanded versions of the WHO ICD-10. Compared to other classifications, the ICD-11 has advantages in terms of concept coverage and compatibility.

Third, the ICD-11 has stronger logical links between codes. If multiple codes are needed for disease expression, there is a lack of practical connections between ICD-10 codes. In the ICD-11, precoordination contains the site and morphology, and the postcoordination of morphology and the clinical phenotype is linked to stem codes through an ampersand (&) and slash (/).

In addition, the ICD-11 contains rich dimensions of postcoordination. Compared to the ICD-10, which provides information only about topography and morphology, the ICD-11 can also include other dimensions of postcoordination, such as stage, grading, laterality, and the diagnostic method. The ICD-11 coding system is more conducive to unifying national cancer registration in these dimensions. However, due to the limitations of the ICD-10 coding structure, these dimensions of postcoordination were missing when mapped to the ICD-11, with only 0.7 postcoordination per code, and its expression ability cannot be truly reflected.

Moreover, the ICD-11 has more expression flexibility. The ICD-10 coding system is fixed and single. The ICD-11 provides 2 sets of codes: ICD-11 MMS codes and URIs. In ICD-11 MMS codes, a stem code can be used alone or with optional extension codes. A URI is a string of characters that uniquely identifies a particular entity. Therefore, a coding system with good expression flexibility can meet the coding granularity and clinical phenotype mining requirements of hospitals at different levels.

Finally, the ICD-11 has different update mechanisms. The ICD-10 updates relatively slowly. In 1999, the WHO established the Update Advisory Committee as the only authoritative body revising the ICD-10. In 2005, the WHO officially published the second edition of the ICD-10 and continued to issue revisions to the relevant content of the ICD-10 through official channels every year. The ICD-11 provides a web-based coding tool instead of paper environments, which is highly beneficial for timely updates. It has a real-time updated orange version and an annually updated blue version.

Overall, the eleventh revision is more extensive than any other revision since the sixth in 1948. These design-level features make the ICD-11 competitive with other classification systems.

### Challenges of Replacement

The improvements described above also pose challenges for replacing the ICD-10 with the ICD-11. Compared to the ICD-10, the ICD-11 leaf codes for neoplasms have expanded by 36.6%, and there are also approximately 16,000 extension codes. Hence, the expression ability of the ICD-11 for clinical details far exceeds that of the ICD-10 and ICD-10-CCM. However, codes with better granularity require clinicians to record diagnoses and treatments in greater detail. It is essential to carry out the necessary interventions to enhance medical record documentation according to ICD-11 before or simultaneously with country-wide implementation [23]. Clinicians will be required to have a good understanding of the ICD-11. Otherwise, the best classification system is just a decoration.

Second, coders are accustomed to using the old classification system and need time to familiarize themselves with the new coding system and tool. Studies have shown that some coders have difficulties coding on the web due to a lack of familiarity with software [24,25]. In this study, 2 skilled coders who received ICD-11 training still had 213 inconsistent codes. Web-based coding requires high professional ability. Studies have also shown that the reliability and accuracy of ICD-11 coding are lower than those of ICD-10 coding [23-25]. In the early stages of ICD-11 promotion, countries involved in the transition need to conduct ICD-11 training and transform coders’ coding concepts. To maintain coding accuracy, developing high-quality ICD-11 training materials, training processes, and local ICD-11 guidelines is effective [23,26].

Moreover, many countries have applied the ICD-10 in different fields, such as mortality and morbidity statistics, diagnosis-related grouping, and cancer registries. There is also a potential challenge with IT systems being able to adopt a new classification that allows for unlimited width in fields. Stakeholders must upgrade their information systems to meet the needs of the ICD-11, which requires a significant amount of time and money to hire medical, IT, and management personnel to complete this transformation. The experience of the United States in replacing ICD versions can provide a better reference. In the United States, because of significant opposition and reservations expressed by stakeholders, it took 6 years from the adoption of the International Classification of Diseases, Tenth Revision, Clinical Modification (ICD-10-CM) to complete the transition [27], and the costs associated with the transition were estimated to be between US $475 million and US $1.5 billion dollars, including training, productivity losses, and system changes [28].

Importantly, the ICD-10 and ICD-11 will coexist for a long time before replacement, which contributes to solving some problems. The parallel implementation of the 2 classification systems can ensure government policy continuity. In January 2022, the pilot application program to promote ICD-11 was launched in China, with 59 large general public hospitals from all provinces participating. However, ICD-11 is not fully understood by other hospitals. The impacts of the new classification system implementation included coding accuracy, ICD version mapping, and more [29]. The ICD version mapping can be used for interoperability between coded data sets [30]. Hospitals do not require dual coding and use ICD-11 to report data. If a hospital needs dual coding, a mapping table can significantly reduce the time, at least by half, spent on duplicate coding. A map can also maintain the same accuracy of ICD-11 coding for all hospitals and alert coders if there are any coding errors. Usually, a high-quality map requires substantial manual curation, and some studies use algorithmic mapping approaches, such as sequential mapping [30]. Some scholars have developed hybrid methods, such as automatic mapping and manual review [31], semiautomatic mapping, and manual evaluation [32]. Although automatic coding has many benefits, supervised and manual mapping are still necessary [33]. Undeniably, there may be some issues with the use of mapping tables. A study has shown that 14.5% of International Classification of Diseases, Ninth Revision, Clinical Modification (ICD-9-CM) codes used by internists, when mapped to the ICD-10-CM, resulted in potential clinical inaccuracies [34]. In general, a high-quality map would contribute to the smooth transition of ICD-11, and multiple mapping methods can be explored. This study confirms the feasibility of using manual mapping tables for neoplasms between the ICD-10-CCM and ICD-11. To date, the results of some studies on the transition to the ICD-11 have also been optimistic [17,18].

In addition, although the WHO provides an ICD-11 coding tool, countries must continue optimizing this tool. A mapping table can improve the intelligence of the coding tool. If the terms are the same as those in the mapping table, the coding tool can automatically load the coding cluster without selecting postcoordination one by one. The accuracy and efficiency of ICD-11 coding largely depend on how well the coding tool is optimized. It is necessary to continuously optimize the coding tool before fully promoting the ICD-11.

### Limitations

This study had some limitations that must be considered. First, this study focused only on the clinical condition of neoplasms. However, the chapter on neoplasms is quite complex and one of the most varied chapters on other conditions, with significant changes. Moreover, patients with cancer may experience different manifestations, including chronic, surgical, and emergency conditions. For example, a patient with long-term chronic hepatitis B causing cirrhosis and liver cancer was admitted to the hospital for emergency surgery due to a tumor rupture. Furthermore, the burden of cancer in China continues to grow with the aging population. According to statistics, the crude cancer mortality rate increased from 108.3 per 100,000 individuals in the 1990-1992 period to 170.1 per 100,000 individuals in 2015 [35]. Significantly, the research method is well thought out. Manual mapping can provide a good understanding of the new ICD-11 features and help individuals familiarize themselves with the characteristics of the new classification systems. As a method, manual mapping can be extended to other conditions. More conditions can be gradually incorporated, especially emergency and surgical conditions, in the future.

Second, this study used only manual mapping, which is time-consuming and laborious. Other methods combined with manual mapping can be continuously explored, especially automatic mapping between the ICD-10-CCM and ICD-11, including algorithmic mapping and machine learning approaches.

### Conclusion

Neoplasm classification has undergone many improvements in the ICD-11, especially the new coding system, improved expression ability, and good semantic interoperability. The new coding system provides advantages in coding granularity, coding capacity, and expression flexibility. Moreover, 67.8% of ICD-10-CCM neoplasm codes can be fully represented by the ICD-11, and expression ability negatively correlates with grouping changes (*r*=–0.144, *P*<.001). The more significant the changes in a new classification system are, the less information can be expressed when mapped to other databases. The use of URIs and maintenance mechanisms can increase the expression ability for coding in actual cases. In addition, the good semantic interoperability of the ICD-11, integrating numerous clinical terminologies from the SNOMED CT, the ICD-O, the tumor node metastasis classification, and expanded versions of the WHO ICD-10, makes the ICD-11 competitive with other classification systems. The transition to the ICD-11 will inevitably bring numerous challenges for clinicians, coders, policy makers and IT technicians, and many preparations will be necessary.

## Abbreviations

ICD-10: International Classification of Diseases, Tenth Revision
ICD-10-CCM: Chinese Clinical Modification of the International Classification of Diseases, Tenth Revision
ICD-10-CM: International Classification of Diseases, Tenth Revision, Clinical Modification
ICD-11 MMS: International Classification of Diseases, Eleventh Revision, Mortality and Morbidity Statistics
ICD-11: International Classification of Diseases, Eleventh Revision
ICD-9-CM: International Classification of Diseases, Ninth Revision, Clinical Modification
ICD-O: International Classification of Diseases for Oncology
SNOMED CT: Systematized Nomenclature of Medicine-Clinical Terms
URI: uniform resource identifier
WHO: World Health Organization
